# MRI-Based Computational Torso/Biventricular Multiscale Models to Investigate the Impact of Anatomical Variability on the ECG QRS Complex

**DOI:** 10.3389/fphys.2019.01103

**Published:** 2019-08-27

**Authors:** Ana Mincholé, Ernesto Zacur, Rina Ariga, Vicente Grau, Blanca Rodriguez

**Affiliations:** ^1^Department of Computer Science, University of Oxford, Oxford, United Kingdom; ^2^Institute of Biomedical Engineering (IBME), University of Oxford, Oxford, United Kingdom; ^3^Division of Cardiovascular Medicine, Radcliffe Department of Medicine, University of Oxford, Oxford, United Kingdom

**Keywords:** clinical MRI-based torso/ventricular anatomical models, computer simulations, electrocardiogram, computational modeling, cardiac magnetic resonance imaging

## Abstract

**Aims:**

Patient-to-patient anatomical differences are an important source of variability in the electrocardiogram, and they may compromise the identification of pathological electrophysiological abnormalities. This study aims at quantifying the contribution of variability in ventricular and torso anatomies to differences in QRS complexes of the 12-lead ECG using computer simulations.

**Methods:**

A computational pipeline is presented that enables computer simulations using human torso/biventricular anatomically based electrophysiological models from clinically standard magnetic resonance imaging (MRI). The ventricular model includes membrane kinetics represented by the biophysically detailed O’Hara Rudy model modified for tissue heterogeneity and includes fiber orientation based on the Streeter rule. A population of 265 torso/biventricular models was generated by combining ventricular and torso anatomies obtained from clinically standard MRIs, augmented with a statistical shape model of the body. 12-lead ECGs were simulated on the 265 human torso/biventricular electrophysiology models, and QRS morphology, duration and amplitude were quantified in each ECG lead for each of the human torso-biventricular models.

**Results:**

QRS morphologies in limb leads are mainly determined by ventricular anatomy, while in the precordial leads, and especially V1 to V4, they are determined by heart position within the torso. Differences in ventricular orientation within the torso can explain morphological variability from monophasic to biphasic QRS complexes. QRS duration is mainly influenced by myocardial volume, while it is hardly affected by the torso anatomy or position. An average increase of 0.12 ± 0.05 ms in QRS duration is obtained for each cm^3^ of myocardial volume across all the leads while it hardly changed due to changes in torso volume.

**Conclusion:**

Computer simulations using populations of human torso/biventricular models based on clinical MRI enable quantification of anatomical causes of variability in the QRS complex of the 12-lead ECG. The human models presented also pave the way toward their use as testbeds *in silico* clinical trials.

## Introduction

The electrocardiogram (ECG) is the most widely used clinical tool for evaluation of cardiac function. It records the electrical activity of the heart from electrodes positioned on the patient’s torso, and the duration, amplitude, and morphology of ECG waveforms in the different leads are used for patients’ diagnosis (Macfarlane and Lawrie, 2010).

Electrocardiogram features, and specifically its QRS complex, are affected not only by microstructural and physiological factors such as fiber orientation, Purkinje, myocardial conduction pathways and ionic currents (Boineau and Spach, 1968), but also by anatomical characteristics such as heart size and orientation, ventricular wall thickness, and body mass index (Hoekema et al., 1999, 2001; van Oosterom et al., 2000; Corlan et al., 2005). Quantitative information on the latter is, however, scarce. An experimental study showed large changes in QRS with varying heart locations, using one isolated perfused dog heart suspended in an electrolytic torso tank (MacLeod et al., 2000). Computer simulation studies are ideally placed to provide insight on the underlying basis of the ECG. Most of the previous computational studies focused on simulating the ECG using a single heart anatomy as (Keller et al., 2010; Zemzemi et al., 2013; Zemzemi and Rodriguez, 2015; Neic et al., 2017; Potse, 2018). More recently, a computational study using torso-biventricular anatomical models for five patients with heart failure showed that heart position and orientation strongly altered QRS amplitude, but only slightly, QRS duration (Nguyên et al., 2015). Sánchez et al. (2018) also provided insights into the key factors determining the ECG characteristics based on data for six heart failure patients. These studies highlight the potential of computer simulation studies using image-based models to shed light into the anatomical basis governing ECG variability and the QRS complex.

Whereas the dense volumetric information and high resolution of current CT scans is a clear advantage in the construction of cardiac anatomical models (Nazarian and Halperin, 2018) for ECG simulations, the radiation involved limits their use, for example in healthy subjects. The alternative of using magnetic resonance imaging (MRI) scans is very attractive as they provide good quality cardiac images safely and non-invasively. Clinical protocols, however, focus on the heart and therefore information on the torso is scarce. This is why previous studies have used MRI scans obtained using dedicated imaging protocols, not suitable for routine clinical practice (Potse et al., 2014; Sánchez et al., 2018). Methodological advances are therefore needed to exploit clinically standard MRI databases in computer simulations studies using image-based human torso/biventricular anatomical models.

The goal of this study is to conduct a computer simulation study using a population of 265 torso-ventricular anatomical models based on clinically standard MRI to dissect and quantify the individual contribution of ventricular and torso anatomy on QRS biomarkers in the 12-lead ECG. We hypothesize that QRS complexes in each of the standard 12-lead ECGs are affected differently by geometrical factors such as ventricular anatomy, heart orientation and location, or torso anatomy. To test this hypothesis, we develop a computational pipeline to conduct high-performance computing (HPC) electrophysiological simulations using biophysically detailed computational human models with ventricular and torso anatomies obtained from clinically standard cardiac MRI acquisitions. In a clinical scenario, the new insights could facilitate an improved discrimination in clinical ECG recordings between the contributions of patient’s anatomical features and those arising from a cardiac condition or disease.

## Materials and Methods

### Reconstruction of Ventricular and Torso Anatomical Meshes From Clinical MRI

**FIGURE 1:**
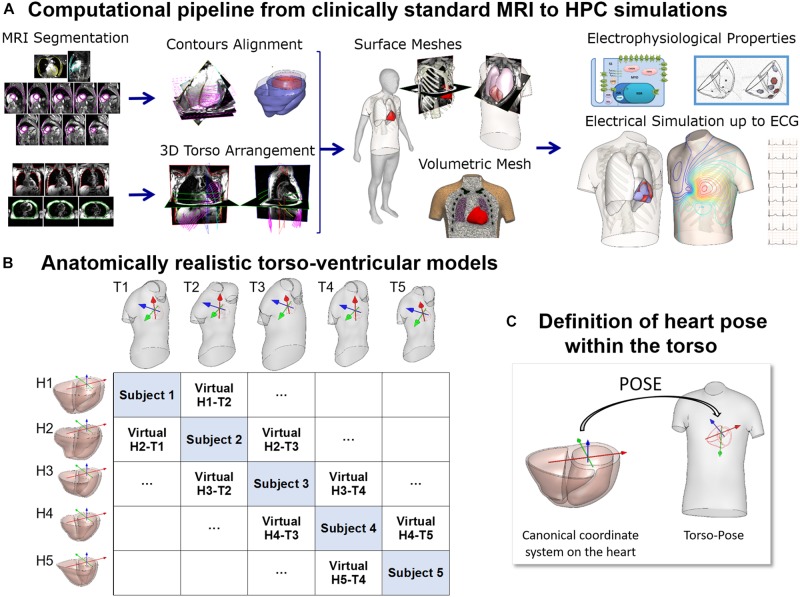
**(A)** Computational pipeline from clinical MRI segmentations through construction of heart and torso geometries to the HPC simulation of electrophysiology from ionic to body surface potentials. Following MRI segmentations, heart surface is obtained by removing breath misalignment and torso surface by using the sparse information from the MRI contours together with a statistical body shape model. With the volumetric meshes, electrophysiological properties such as an action potential model and an activation model are used to simulate electrical activity from cell to torso and calculate the 12-lead ECG. **(B)** 25 torso-ventricular anatomical models combining five torsos (T1–T5) and five ventricles (H1–H5) of varying volumes. **(C)** Heart pose within the torso defined as the transformation from a canonical coordinate system of the ventricular geometry to the torso coordinate system.

In this study, a total of 265 combined torso-ventricles anatomical models were considered to quantify the effect of ventricular and torso volumes, and heart position and orientation on the QRS complex. Initially, as described in Figure 1, twenty-five human heart-torso models were generated by combining the bi-ventricular geometries (H1–H5) and torsos (T1–T5) (including corresponding heart orientations and positions) extracted from clinical MRI acquisition from 5 healthy subjects. The MRI datasets were selected to include ventricular end diastolic myocardial volumes between 75 and 170 cm^3^ and torso volumes between 23 and 54 dm^3^. Then, either rotation or translation was applied to each bi-ventricular model within each torso. 5° steps up to (±40° were considered both around the long axis (LA) and around the left-to-right-ventricle axis (LR) (Nguyên et al., 2015). Translation was considered in 1 cm steps up to (±4 cm either along the lateral or along cranio-caudal directions.

The MRI scans were obtained in five healthy subjects (three females and two males) with a range of ventricular end-diastolic myocardial volumes between 75 and 170 cm^3^ and torso volumes between 23 and 54 dm^3^, recruited at John Radcliffe Hospital, Oxford, United Kingdom. Subjects were non-smokers without cardiovascular disease, hypertension or diabetes, and no family history of cardiomyopathy or sudden cardiac death (SCD). Clinically standard cine cardiac MRI acquisition was performed for each subject, including long axis (LAX) and a stack of short axis (SAX) views. More specifically, for each subject, the data includes a 2 chamber LAX view, 4 chamber view, and a stack of SAX view from apex to base with 10 mm of separation between adjacent slices in the stack (8 mm slice thickness plus 2 mm gap). Image resolution ranges from 1.4 to 1.6 mm per pixel. An expert with several years of experience in cardiac MRI segmented the images at end-diastole including the following structures: left epicardium, left ventricle (LV) endocardium excluding the papillary muscles, and right ventricle (RV) endocardium (see Figure 1A, Segmentation). As the image resolution in standard MRI acquisitions does not allow to differentiate right ventricular epicardial and endocardial contours in the right ventricle, we synthesized right epicardial contours by a 3.5 mm offset from the endocardial contours (Prakash, 1978). Spatial misalignments in slice images and spatial discrepancies between the contours due to acquisitions at different breath holds were corrected by aligning intensity profiles of intersecting slices using a 3D rigid transformation for each image (Villard et al., 2017) (see Figure 1A, Contours alignment). Bi-ventricular geometries were built from the aligned contours using the end-diastole frames from the standard CINE acquisition as in Villard et al. (2018) and Zacur et al. (2017).

For the construction of the torso geometries, semi-automatic tools were developed and used to delineate the torso skin and lungs (Zacur et al., 2017). In brief, on each subject, the scout images (localizers), as well as, most of the MRI images (SAX and LAX images) with a large enough field of view were used and contoured. The sparse 3D geometrical information from the torso images is insufficient for the use of classical segmentation to surface methods such as isocontouring tools (Figure 1A, 3-Dimensional torso arrangement). Thus, we developed and applied a methodology to fit a statistical shape model of the human body to the skin contours (Zacur et al., 2017). The torso contours together with subject height, weight and gender information were used to reconstruct a body surface belonging to a learned class of plausible body shapes from the statistical shape model (Pishchulin et al., 2017; Zacur et al., 2017). The average discrepancy between MRI-based contours and model surface is 3 mm in terms of root mean square, being the 90th percentile 5 mm. A template-based approach was used to place other internal structures such as the lungs and the ribs (Figure 1A, Surface meshes). Full details about the procedure are provided in Zacur et al. (2017).

All surfaces were remeshed with different element sizes to ensure numerical convergence of the finite element software Chaste for electrophysiological simulations (Pitt-Francis et al., 2009; Dutta et al., 2016) as described in Supplementary Material S1. Finally, tetrahedral volumetric meshes were constructed from these surfaces (see Supplementary Material S1). The surface and volumetric meshes can be downloaded from http://www.cs.ox.ac.uk/ccs/home.

Figure 1B shows the reconstructed ventricular geometries and, the torso geometries including their corresponding heart orientations and positions, constrained by chest boundaries. The generation of a new virtual torso-ventricular geometry requires the transformation of the ventricular geometry from a canonical reference frame to the position defined by the torso (pose, see Figure 1C). Hereinafter, torso-pose is defined as the torso geometry including the heart pose, which represents the coordinate system and location of the heart. This torso-pose linking is supported by physical constraints such as chest boundaries, since one specific torso anatomy does not allow any heart position (Engblom et al., 2005). Further information is found in Supplementary Material S1.

### Electrophysiological Simulations

The anatomical torso-ventricular model combinations described above were used to compute 265 QRS complexes from computer simulations as follows. The propagation of the electrical activity in the human ventricles and torso was modeled using the fully coupled heart-torso bidomain equations and solved with the Chaste software (Pitt-Francis et al., 2009). Human ventricular membrane kinetics were simulated with a modified version of the O’Hara-Rudy action potential model (O’Hara et al., 2011) published in Dutta et al. (2017). Myocardial and torso conductivities, and, myocardial fiber structure were set as described in Supplementary Material S2. An anisotropic myocardial fiber architecture was implemented using the Streeter rule (Streeter et al., 1969). The three orthotropic intracellular and extracellular myocardial conductivities were set as in Cardone-Noott et al. (2016). Transmural, apex-to-base and interventricular cell electrophysiological heterogeneities were introduced based on experimental and clinical data and as described in Supplementary Material S2. The QRS complex is hardly affected by the electrophysiological heterogeneities included in our models as they mainly affect the repolarization properties and the T wave. However, they provide our computational pipeline with all the state of the art capabilities to extend the work to investigate variability in T wave morphology, as well as under disease and drug action.

Sinus rhythm was simulated using a phenomenological activation model with early endocardial activation initiated by root nodes and a fast endocardial layer representing a tightly packed endocardial Purkinje network (Cardone-Noott et al., 2016). In short, 7 root nodes are positioned in the ventricles on the endocardium: four in the LV (LV mid septum, LV anterior paraseptal, and two LV mid-posterior) and three in the RV (RV mid septum, two RV free wall), as shown in Supplementary Material S2. Simulated ventricular activation times for these models show the LV endocardial surfaces are fully activated within a range of 39 to 51 ms, and the latest moments of activation occurs in a range from 57 to 76 ms. This is in agreement with the *ex vivo* microelectrode recordings by Durrer et al. (1970) reporting around 45 ms in endocardial LV activation, and from 60 to 80 ms the latest moments of whole ventricular activation.

**FIGURE 2:**
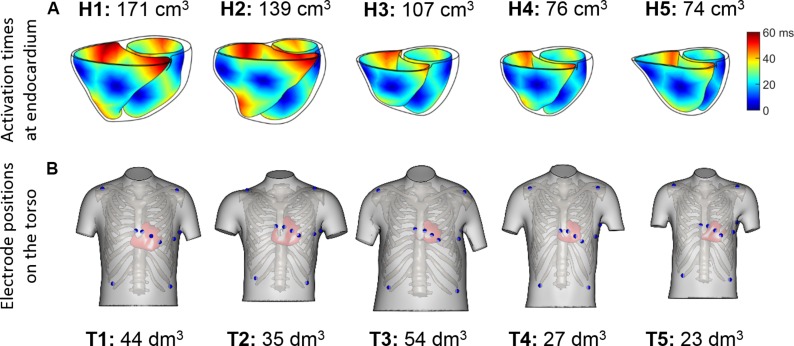
**(A)** Endocardial activation maps for the five ventricular geometries. Information about ventricular volumes is also provided. **(B)** Homologous 12-lead electrode positions for the five virtualized subjects. Information about the torso volumes is provided.

The aim of this study is to investigate, analyze and quantify the effect of anatomical/geometrical variability on the QRS complexes in an electrophysiological computer simulation framework. It is not to construct personalized electrophysiology models to replicate each of the patients’ data. In order to isolate the effect of ventricular anatomy from differences in activation patterns, the endocardial speed was set to 120 cm/s in all models, and the locations of the root nodes were mapped to anatomically homologous locations from the geometry used in a previous study (Cardone-Noott et al., 2016). The coupled epicardial, RV and LV endocardial surfaces and ventricular insertion points from the original geometry (Cardone-Noott et al., 2016) were diffeomorphically registered to each ventricular geometry using a composition of approximated Thin-Plate Splines (TPS) deformations (Rohr et al., 2001). The deformation method was guided by an iterated closest point between the corresponding source and target surfaces/structures (left and right endo- and epicardial surfaces, atrio-ventricular planes, and anterior and posterior interventricular grooves). The successive deformations were performed by following an annealing process in the smoothness parameter of approximated TPS (Amberg et al., 2007). The resulting registered deformation was applied to the early activation sites from the original geometry resulting in the anatomically homologous locations that lead to similar activation sequences (see Figure 2A). The resulting deformations and the mapped activation sites were visually evaluated and approved by an expert cardiologist. This technique was used given its broad acceptance and success in the medical imaging and shape analysis field but the universal ventricular coordinates could represent an alternative (Bayer et al., 2018).

QRS complexes from the 12-lead ECGs were simulated by placing virtual electrodes in the standard 12-lead ECG positions for each torso. Since the statistical shape model used for reconstructing the torsos is based on anatomical correspondences, the virtual electrodes are located in anatomically homologous locations for all the torsos (see Figure 2B). These electrode positions correspond to analogous intercostal spaces for all subjects. Electrode coordinates are given for each of the torso geometries, and can be downloaded from http://www.cs.ox.ac.uk/ccs/home.

To simulate the QRS complex for 265 combinations of ventricular/torso positions and orientations while minimizing the number of expensive HPC simulations, extracellular potentials were computed from the ventricular potentials following the integral of dipole source density formulation (Gima and Rudy, 2002; Plonsey and Barr, 2007) as:

$$\phi \,\left(e\right)\,\text{=}\,\int_{\text{Ω}}\left(-D\,\nabla V_{m}\cdot \left(\nabla \frac{1}{||r-e||}\right)\right)\,d\,r,$$

where *e* = (*e*_*x*_,*e*_*y*_,*e*_*z*_) are the electrode position coordinates, *D* is the diffusion tensor, and *V*_*m*_ is the membrane potential. The integral is calculated over the whole myocardium volume,Ω. Bidomain simulations coupled with the Poisson equation to propagate the electrical activity to the body surface were compared to the integration of dipole source density formulation for the torso propagation. The resulting QRS complexes were very similar as shown in Supplementary Figure S3 from Supplementary Material S2. The propagation model based on the integration of dipole source density formulation was chosen in order to simplify the numerical complexity of the computations and to avoid re-meshing the torso volume for each scenario in which the ventricular geometry was rotated, translated or permuted. Although this method does not allow the inclusion of tissue inhomogeneities in the torso, several studies suggest minimal differences in the resulting body surface potentials and the QRS complex when assuming homogeneous or inhomogeneous torso models (Ramanathan and Rudy, 2001; Geneser et al., 2008).

### Quantification of QRS-Based Features and Descriptors

Clinically used features from the QRS, such as duration and amplitude, were extracted for each of the simulated 12-lead ECGs. QRS duration is calculated by using a relative threshold on the absolute value of the slopes of the ECG signal to identify QRS onset and offset as in Martínez et al. (2004). We compared the simulation results with those reported in the literature and also clinical ECG recordings from healthy volunteers, as part of a prospective study approved by the National Research Ethics Committee (REC ref 12/LO/1979). Informed written consent was obtained from each participant (Lyon et al., 2018a).

Furthermore, in order to quantify the effect of anatomical variability in QRS morphology, we proposed a similarity measure to quantify QRS morphological differences, invariant to QRS amplitude and duration. The new metric (PC^∗^) is based on a continuous generalization of the Pearson coefficient (PC), which in order to ensure independence from QRS duration, includes the invariance to a uniform warping (time scaling) in time of the QRS complex. Therefore, PC^∗^ between two QRS complexes is 1 when these have the same morphology regardless of the amplitude or the duration.

Let *f* and *g* be two functions defined on the domains $\text{dom}\,\left(f\right)\,\text{=}\,\left[t_{\text{0}\;}^{f},t_{\text{1}\;}^{f}\right]$ and $\text{dom}\,\left(g\right)\,\text{=}\,\left[t_{\text{0}\;}^{g},t_{\text{1}\;}^{g}\right]$, respectively. Let’s assume that dom(*f*)∩dom(*g*)≠∅ and let’s consider the combined domain [*t*_0_,*t*_1_]=dom(*f*)∪dom(*g*), where $t_{\text{0}\;}\,\text{=}\,\min\left(t_{\text{0}\;}^{f},t_{\text{0}\;}^{g}\right)$ and $t_{\text{1}\;}\,\text{=}\,\max\left(t_{\text{1}\;}^{f},t_{\text{1}\;}^{g}\right)$. On this combined domain, let *f̃* be the replicated extension of the original function  *f*,

$$\,\tilde{f}\,\text{=}\,\left\{ \begin{array}{c} f\,\left(t\right)\;\text{if}\,t\in \left[t_{\text{0}\;}^{f},t_{\text{1}\;}^{f}\right]\\ f\,\left(t_{\text{0}\;}^{f}\right)\;\text{if}\,t<t_{\text{0}\;}^{f}\\ f\,\left(t_{\text{1}\;}^{f}\right)\;\text{if}\,t<t_{\text{1}\;}^{f} \end{array}\right.$$

and equivalently for *g̃*.

Our proposed generalized PC is given by

(1) $${\text{PC}}_{f,g}\,\text{=}\,\frac{1}{t_{\text{1}\;}-t_{\text{0}\;}}\,\int_{t_{\text{0}\;}}^{t_{\text{1}\;}}\frac{\tilde{f}\,\left(t\right)-\mu_{\tilde{f}}}{\sigma_{\tilde{f}}}\cdot \frac{\tilde{g}\,\left(t\right)-\mu_{\tilde{g}}}{\sigma_{\tilde{g}}}\,d\,t$$

where $\mu_{\tilde{f}}$ and $\mu_{\tilde{g}}$ are means, and $\sigma_{\tilde{f}}$ and $\sigma_{\tilde{g}}$ are the standard deviations of the functions *f̃*(*t*) and *g̃*(*t*), respectively,

$$\mu_{\tilde{f}}\,\text{=}\,\frac{1}{t_{\text{1}\;}-t_{\text{0}\;}}\,\int_{t_{\text{0}\;}}^{t_{\text{1}\;}}\tilde{f}\,\left(t\right)\,d\,t,\sigma_{\tilde{f}}\,\text{=}\,\sqrt{\frac{\text{1}}{t_{\text{1}\;}-t_{\text{0}\;}}\,\int_{t_{\text{0}\;}}^{t_{\text{1}\;}}{\left(\tilde{f}\,\left(t\right)-\mu_{\tilde{f}}\right)}^{\text{2}\;}\,d\,t}$$

and equivalently for $\mu_{\tilde{g}}$ and $\sigma_{\tilde{g}}$. The subtraction of the means $\mu_{\tilde{f}}$ and $\mu_{\tilde{g}}$ in Eq. (1) ensures the invariance of PC under changes in the baseline levels. Likewise, normalizations by $\sigma_{\tilde{f}}$ and $\sigma_{\tilde{g}}$, endow PC with invariance to scaling. The normalization by (*t*_1_-*t*_0_) allows independence from time units. Thus, PC values are within the [–1, 1] interval.

In order to ensure independence from QRS duration, we include the invariance to a uniform warping (time scaling) in time through the following similarity measurement PC^∗^:

$${\text{PC}}_{f,g}^{*}\,\text{=}\,\max_{s\in {\mathbb{R}}^{+}}{\text{PC}}_{f\left(\cdot \right),g\left(s\cdot \right)},$$

where *g*(*s*⋅) is a uniformly time-warped version of *g*. It is worth mentioning that since $\text{dom }\left(g\left(s\cdot \right)\right)\text{=}\text{1}/s\cdot \text{dom}\left(g\right)\text{=}\left[t_{\text{0}\;}^{g}/s,t_{\text{1}\;}^{g}/s\right]$, the integral interval in Eq.(1) is updated accordingly, and the resulting PC^∗^ keeps having comparable values.

In the following, we will explain how to quantify a global similarity for a set of *N* functions {*f*_1_ (⋅), *f*_2_ (⋅), ⋯, *f*_*N*_ (⋅)}. We propose to compute, the best time warping factors for the N functions simultaneously, in order to optimally align the set. Therefore, we search for

$$\left\{s_{\text{1}\;},s_{\text{2}\;},\ldots ,s_{N}\right\}\,\text{=}\,\underset{s_{\text{1}\;},\ldots ,s_{N}\in {\mathbb{R}}^{+}}{\text{argmax}}\underset{\begin{array}{l} \;i=1\ldots N\\ j=i+1\ldots N \end{array}}{\min}{\text{PC}}_{f_{i}\left(s_{i}\cdot \right),f_{j}\left(s_{j}\cdot \right)}$$

Once these optimal time warping factors have been computed, the global similarity for the functions {*f*_1_(⋅),*f*_2_(⋅),⋯,*f*_*N*_(⋅)} is given by

$${\text{PC}}_{f_{\text{1}\;},f_{\text{2}\;},\ldots \,f_{N}}^{*}\,\text{=}\,\underset{\begin{array}{l} \;i=1\ldots N\\ j=i+1\ldots N \end{array}}{\max}{\text{PC}}_{f_{i}\left(s_{i}\cdot \right),f_{j}\left(s_{j}\cdot \right)}$$

with this, the worst aligned pair defines the similarity of the whole set.

## Results

### Effect of Ventricular Geometry on QRS Duration and Amplitude

**FIGURE 3:**
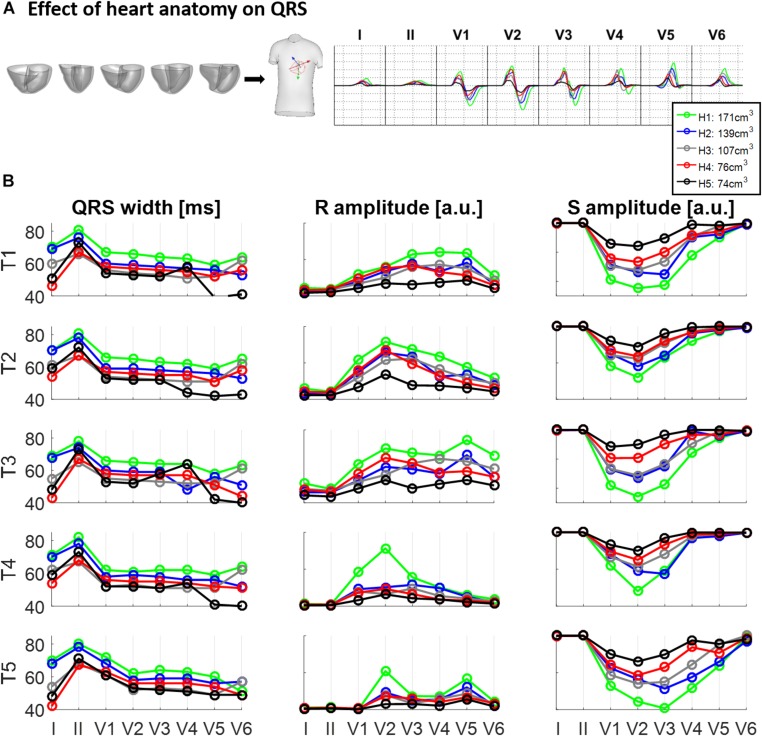
Effect of ventricular geometry on QRS duration, and S and R wave amplitude. **(A)** Simulated QRS complexes obtained for five ventricular geometries placed in the torso-pose of Subject 3 (T3). **(B)** QRS duration (left), S and R amplitude (middle and right, respectively) obtained from simulations using the five ventricular geometries (H1 to H5) placed in each of the five torso-poses (T1 to T5). The term “a.u.” stands for arbitrary units.

Figure 3 illustrates the effect of different ventricular geometries within the same torso with corresponding heart position (hereinafter referred to as torso-pose) on QRS duration and amplitude. Figure 3A shows the QRS complexes obtained in leads I, II and V1–V6 for each of the five ventricles (H1–H5) placed in the torso-pose of Subject 3 (T3). Figure 3B shows QRS complex duration as well as S and R wave amplitude for all 25 torso-pose and ventricular combinations. For all torso-poses, QRS complexes decrease in amplitude and increase in duration with an increase in myocardial volume (green versus black traces corresponding to the largest versus the smallest ventricular volumes, respectively). An average increase of 0.12 ± 0.05 ms in QRS duration for each cm^3^ of myocardial volume across all the leads was found. Relationships between QRS durations and ventricular myocardial volumes for each of the leads is shown in Supplementary Material S4. Supplementary Figure S4 shows the 12 lead QRS complexes of different ventricular geometries within the same torso-pose for each of the five subjects.

### Effect of Torso-Heart Position on QRS Duration and Amplitude

**FIGURE 4:**
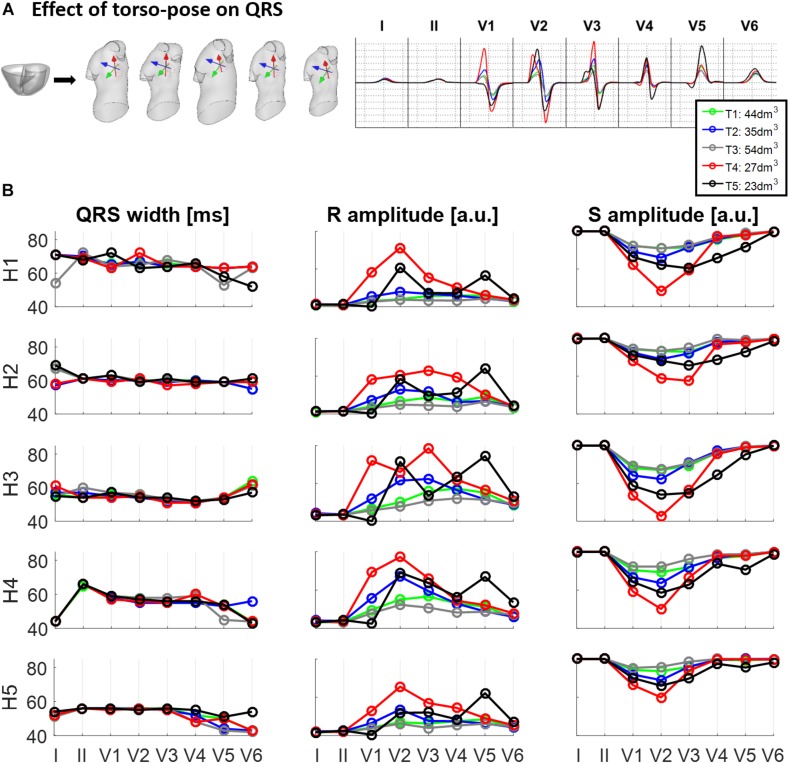
Effect of torso-pose on QRS duration, and S and R wave amplitude. **(A)** Simulated QRS complexes obtained using the ventricular model from Subject 3 (H3) placed in the five different torsos-poses. **(B)** QRS duration (left), and S and R amplitudes (middle and right, respectively) from simulations using each of the five hearts (H1 to H5) placed in the different torso-poses (T1 to T5).

Figure 4 shows the effect of different torso-poses on QRS duration and amplitude. Figure 4A shows as an example, the QRS complexes obtained for the ventricular geometry H3 when placed in all the torso-poses, and Figure 4B provides quantification of QRS duration and R and S wave amplitudes for the five ventricular geometries. QRS duration does not change substantially for different torso-poses, and this suggests that QRS duration is mainly determined by the ventricular geometry. Indeed, a slight increase in QRS duration of 0.01 ± 0.03 ms for dm^3^ of torso volume across all leads is observed (further information regarding the relationship between QRS duration and torso volume can be found in Supplementary Material S4). However, both S and R wave amplitudes are mainly determined by torso volumes, with larger QRS amplitudes corresponding to smaller torso volumes. Exceptionally, QRS complexes in T4 with a torso volume of 27 dm^3^ exhibit larger amplitudes in V1 to V3 compared to T5 (23 dm^3^) that exhibit larger amplitudes in V4 to V6. For these two torsos with similar volumes, heart position plays an important role in QRS amplitude. By comparing the ventricular positions for T4 and T5 (see Figure 2), we observe that for T5, V5 and V6 electrode positions are closer to the ventricles resulting in larger QRS amplitudes, whilst V2 and V3 electrodes are further away, resulting in smaller amplitudes.

### Effect of Ventricular Geometry and Torso-Pose on QRS Morphology

**FIGURE 5:**
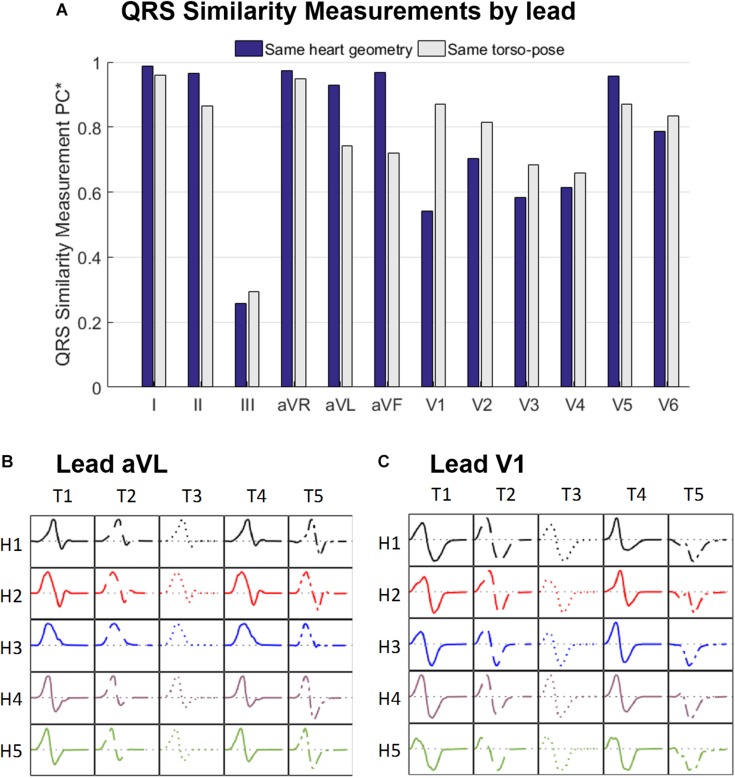
**(A)** Similarity measurement (PC^∗^) of the simulated QRS morphologies for fixed ventricular geometry (and varying torso-pose) (blue), and for fixed torso-pose (and varying ventricular geometry (gray). Panels **(B,C)** Simulated QRS morphologies obtained with the five ventricular geometries (H1 to H5) placed in the five torso-poses (T1 to T5) for leads aVL **(B)** and V1 **(C)**.

Figure 5A displays the similarity measurement computed from the modified Pearson correlation PC^∗^, which measures differences in QRS morphology due to differences in ventricular geometry and torso-pose.

Results show that for limb leads (I, II, aVR, aVL and aVF), and V5, QRS morphology is more similar (and PC^∗^ higher) for fixed ventricular geometry (with varying torso-pose) than for fixed torso-pose (with varying ventricular geometry). Therefore, in these leads, the ventricular geometry mainly determines the QRS morphology.

On the contrary, for leads V1 to V4 and V6, QRS morphology is more similar (as shown by the higher PC^∗^ values) for fixed torso-poses than for fixed ventricular geometry. Thus, in these leads, QRS morphology is mostly determined by torso-pose rather than by ventricular geometry.

These results are further illustrated in Figures 5B,C for two representative leads, aVL and V1. Simulated QRS morphology is mostly determined by the ventricular geometry and torso-pose in leads aVL and V1, respectively. Simulated QRS complexes obtained with the same ventricular geometry are shown in the same row while those obtained with the same torso-pose are shown in the same column. The warped QRS complexes from which PC^∗^ is computed are shown in Supplementary Material S5.

### Effect of Heart Orientation and Position on the QRS Morphology

**FIGURE 6:**
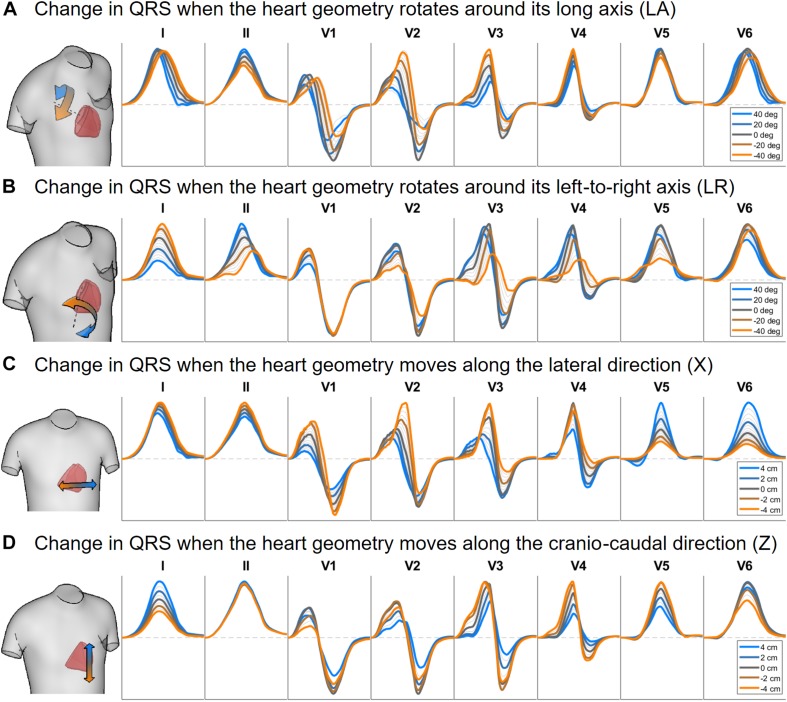
Simulated QRS complex in leads I, II and V1 to V6 for different rotation angles around the long axis **(A)** and the left-to-right-ventricle direction **(B)**, and for different heart translations along the lateral **(C)** and cranio-caudal **(D)** directions. Gray lines correspond to intermediate results showing how the signals evolve from one colored state to another. The heart and torso geometries correspond to subject 3.

**FIGURE 7:**
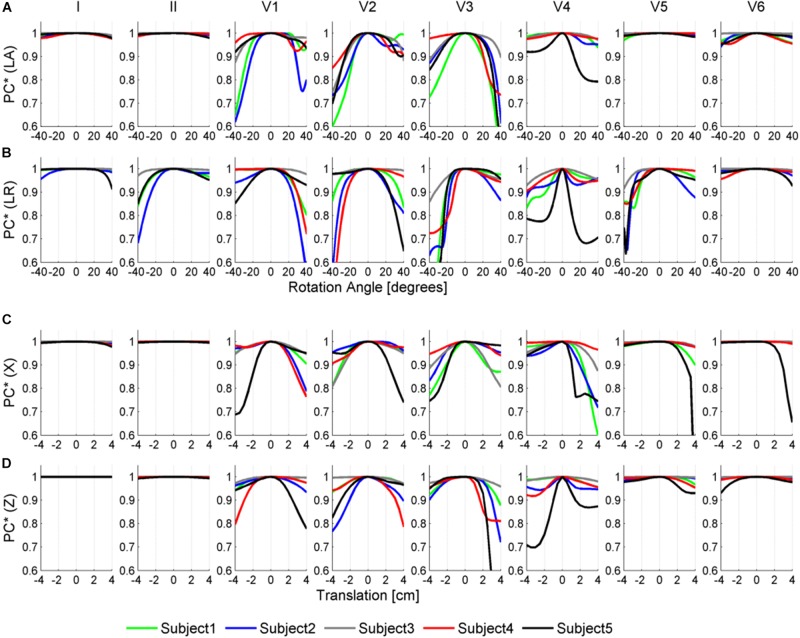
Similarity measurement (PC^∗^) for each lead (I, II, V1–V6) between the QRS at the original pose and the QRS when the heart is rotated along the long axis (LA) in panel **(A)**, the left-to-right-ventricles axis (LR) in panel **(B)**, and when translated in the lateral and cranio-caudal directions in panels **(C,D)**, respectively. Rotation angles range from –40° to 40° and translations range from –4 to 4 cm.

Figures 6, 7 illustrate the results obtained from the 265 simulations conducted to evaluate the effect of rotation around the long axis and left-to-right ventricle directions and translation along the lateral and cranio-caudal directions of the ventricles within the torso in the QRS complex. Figure 6 displays simulated QRS complexes obtained for a representative anatomical model (H3 within T3), whereas Figure 7 shows quantification of the QRS morphology similarity metric (PC^∗^) for all subject specific torso-ventricular geometries.

As shown in Figures 6A, 7A, rotation along the long axis mainly affects the R and S amplitudes of leads V1 to V3. The amplitude of the R wave is larger when the LV faces the chest plane (orange traces) and decreases as the RV gets positioned between the LV and the chest (blue trace). The amplitude of the S wave also decreases in the precordial leads in ventricular positions where the RV faces the chest. Quantitative results regarding changes in QRS amplitudes with heart orientation and position can be found in Supplementary Material S6.

Figures 6B, 7B show that rotation around the left-to-right ventricle axis severely affects the QRS morphology in leads II, and V1 to V5. More horizontal hearts (orange traces) result in larger R wave amplitudes in leads I, V1, and V6 while more vertical ones (blue traces) result in larger R and S wave amplitudes in leads V2 to V5.

Figures 6C, 7C show that hearts located in more medial positions result in taller R and S waves in septal V1 to V3 leads while shorter R waves are observed in the precordial lateral leads V5 and V6. This is due to the closer ventricular location to V1 to V3 electrode positions and further from V5 and V6. On the other hand, hearts located in more lateral positions (toward the left-arm) displayed negatively deflected rS or even QS complexes in the septal V1 to V2 leads and larger R wave amplitudes in V5 to V6 (blue solid lines).

Figures 6D, 7D show the effect of shifting the ventricles up along the cranio-caudal (superior-inferior) direction leads to larger R wave amplitudes in lead I, and longer R and S waves in V1 to V5 (blue lines). However, shifting the heart down leads to shorter R waves in leads I, V1 and V6 (orange line). The changes would be equivalent to changing the electrode position with respect to the ventricles.

### Comparison to Clinical Data

Simulated ECGs obtained from our population of models exhibit QRS axis (computed from QRS complexes in leads I and III) ranging from 50° to 75° [normal range –30° to 90° as shown in Engblom et al. (2005)], QRS durations per lead from 45 to 80 ms [normal range including all leads 78 ± 8 ms as shown in van Oosterom et al. (2000)], and amplitudes from 0.5 to 3.5 mV [healthy: 2 ± 0.6 mV as shown in van Oosterom et al. (2000)]. Thus, all these quantitative measurements are in agreement with clinical ECGs from healthy subjects (van Oosterom et al., 2000; Engblom et al., 2005; Stewart et al., 2011), supporting the credibility of the simulations.

**FIGURE 8:**
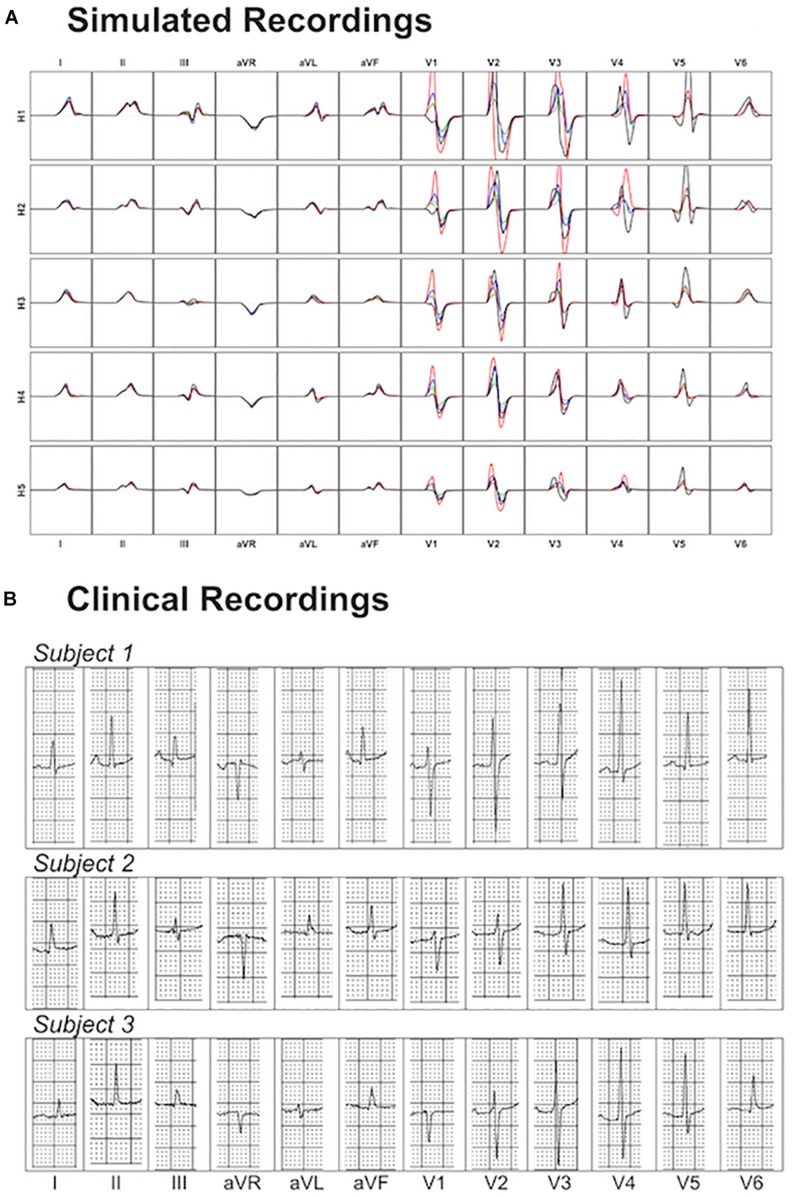
Comparison between simulated and clinical 12-lead ECG recordings. Panel **(A)** shows the simulated 12-lead QRS complexes obtained using each of the ventricular models (H1–H5) placed in the five different torsos-poses. Panel **(B)** shows three exemplary clinical 12-lead ECG recordings from healthy subjects.

Figure 8 shows a comparison of the variability exhibited in simulated and clinical 12 lead ECG QRS complexes. Simulated QRS complexes show variability in terms of morphology, especially in the precordial leads. The normal upright (positive) QRS complexes in both, lead I and lead aVF, result in a normal QRS axis. Furthermore, simulated QRS complexes show positive deflection with large, upright R wave in leads I, II, V4–V6 and a predominant negative deflection with a large, deep S wave in aVR, V1 and V2 (see Figure 8A). This is in agreement with the three clinical recordings shown in Figure 8B. Lead III in simulated ECGs shows biphasic QRS complexes with a negative deflection followed by a positive one as in the clinical recording of Subject 1 (Figure 8B). On the contrary, simulated lead aVL shows biphasic QRS complex with first a positive deflection followed by a negative one, as in clinical recordings of Subjects 1 and 3 (Figure 8B).

Precordial QRS complexes show R wave progression from V1 to V6, with an increasing R wave and a decreasing S wave when moving from V1 to V6. This progression is observed in both simulated (Figure 8A) and clinical ECGs (Figure 8B). QRS complexes in lead V1 show morphological variability from biphasic QRS complexes (positive-negative deflections) to downright QRS complexes (Figure 8A). This is in agreement with the variability in clinical recordings (Figure 8B).

## Discussion

The present study demonstrates the computational evaluation of the effect of heart-torso position and anatomy on the QRS complex using human torso/biventricular electrophysiology models derived from clinically standard MRI. The first contribution of the study is the computational pipeline to build the torso/biventricular anatomies initiating from standard clinical cardiac MRI augmented with a statistical shape model of the body (Zacur et al., 2017). This methodology enables exploiting clinical databases to evaluate the functional impact of MRI-extracted anatomical and structural features (Lyon et al., 2018b). Furthermore, human MRI-informed modeling and simulation based on this technology could accelerate the development of tailored pharmacological and electrical therapy and the goal for precision care. Firstly, being able to reconstruct the patient’s specific torso and ventricular model from standard cardiac MRI is a step forward to personalized computer modeling and simulation. The human models constructed have the biophysical detail required to enable future simulation studies into the response to disease and pharmacological treatment. Additionally, the simulation results demonstrate the influence of anatomical features on the QRS complex in healthy control conditions. This quantification of normal QRS complex variability is important to inform the evaluation of response to disease and treatment.

Analysis of the simulated QRSs yields the following findings: (i) QRS morphologies in limb leads I and II are mainly determined by the geometry of the ventricles whereas QRS morphologies in the precordial leads, and especially V1 to V4, are determined by the torso-pose. (ii) QRS duration is mainly influenced by myocardial volume while it is hardly affected by the torso geometry or heart position; (iii) QRS amplitude increases with large ventricular volumes and decreases with larger torso volumes. Quantification of the contribution of the individual heart structure, orientation and body habitus on the ECG is critical to aid the clinical interpretation of potential ECG abnormalities driven by disease or pharmacological treatment. In synergy with clinical databases, they could also drive the personalization of score metrics for risk stratification by discriminating in clinical recordings between the contribution of each patient’s specific anatomy and those arising from their disease state.

### Populations of Heart-Torso Electrophysiological Models From Standard Clinical MRI

In this paper, we present multiscale electrophysiological simulations using heart-torso anatomical models from standard cardiac MRI acquisitions (Figure 1). The generation of the subject-specific geometries is performed from standard cardiac MRI acquisitions allowing to be used directly on available clinical datasets. The scarce information of torso anatomy from standard cardiac MRI acquisitions makes the use of traditional image-to-surface methods difficult and therefore, previous studies have required the use of dedicated imaging protocols (Potse et al., 2014). In the present pipeline, we make use of a statistical shape model together with the MRI information to accurately build the patient-specific torso geometry (Pishchulin et al., 2017; Zacur et al., 2017).

The comprehensive computational pipeline connects the outputs and inputs of different technologies, including the generation of torso-ventricular geometrical surfaces, the tetrahedralization into a multimaterial volumetric mesh, the generation of fiber orientations in the myocardium, the different input files to be used in the Chaste software to derive the fiber orientations and the endocardial layer for fast stimulation mimicking the Purkinje system, to assign different electrophysiological cell properties and finally to simulate the electrical activity and the 12-lead ECG. All files and torso-ventricular geometrical meshes are available for future studies.

In the evaluation of the role of anatomical features on the ECG, previous studies have typically performed perturbations to anatomical features (i.e., heart positioning and orientation) around a reference/original position to explore their individual effect (Corlan et al., 2005; Nguyên et al., 2015). In the present study, we sample the space of plausible anatomies and generate plausible instances by swapping ventricular anatomies together with torso-poses using their corresponding heart position and orientations (torso-poses) (see section “Reconstruction of Ventricular and Torso Anatomical Meshes From Clinical MRI”). This approach allows us to explore the role of ventricular anatomy on the QRS complex without the need to define a high dimensional parametrization of the ventricular shape. In particular, this population provides a generic open framework for further studies aimed to explore how anatomy and its variability influence the signature of the cardiac function in the ECG.

### QRS Morphologies Are Determined by Ventricular Geometry in the Limb Leads and by Heart Orientation in Septal Leads

Ventricular geometry and not the torso-pose mainly determines the QRS morphology in the limb leads I and II, augmented leads, and V5 (see Figure 5). This finding may explain the weak correlation found in a number of studies between the anatomical heart orientation and the cardiac electrical axis (Dougherty, 1970; Horan, 1987; Engblom et al., 2005; Pellicori et al., 2015; Sathananthan et al., 2015). Cardiac electrical axis (Kashou and Kashou, 2018) is mainly estimated using the QRS complexes from leads I, II and aVF which, as shown in Figures 5, 7, are more influenced by ventricular geometry than by heart orientation. Therefore, this results in a lower correlation between the electrical and anatomical axis.

On the other hand, QRS morphology in septal and anterior leads V1 to V4, III and V6 is mostly determined by the torso anatomy and its linked heart positioning. This could also result from changes in electrode position. The two different sources of variation, ventricular anatomy and torso-pose, affecting mainly limb/augmented leads and precordial leads, respectively, may have implications on the normal variability of QRS morphology in the different leads.

The proposed QRS similarity measurement is a global morphological measurement able to quantify differences in the QRS morphology due to the effect of anatomical variability. Unlike other studies proposing feature based differences as for example (Giffard-Roisin et al., 2017; Sánchez et al., 2018), we have measured differences in QRS morphology complementary and independent of QRS duration and amplitude. Our quantification allows a lead-wise evaluation of the influence of ventricular anatomy and torso-pose on the QRS morphology.

Importantly, the results on the contribution of the ventricular anatomy and the torso-pose on the QRS morphology of each of the leads do not depend on the endocardial and myocardial conduction (see Supplementary Material S7).

### QRS Duration Depends Primarily on Ventricular Size and Is Hardly Affected by Heart Pose and Orientation

This study shows a positive correlation between ventricular volume and QRS duration (see Figure 3). Even if this finding is intuitive, little data exist on the influence of heart size and body weight on QRS duration. This correlation may have implications for the normal ranges of QRS duration and thus on the diagnosis of QRS prolongation. Most of the studies relating QRS duration and ventricular mass are in the context of patients at high cardiovascular risk (Stewart et al., 2011) and do not consider control subjects. Therefore, many factors may intervene for long QRS duration such as a reduced LV ejection fraction (Murkofsky et al., 1998) or ventricular dys-synchrony that are unrelated to LV hypertrophy (Oikarinen et al., 2004). On the contrary, we show that the torso-pose hardly modifies the QRS duration for any of the ventricular geometries (see Figure 4). This is in agreement with (Nguyên et al., 2015) that quantifies QRS duration differences from –6 to 10% in five heart failure patients.

### Both Torso and Heart Geometrical Factors Have a Role in the QRS Amplitude

Ventricular and torso volumes are generally related since large body masses usually correspond to large hearts. Indeed, a recent paper in the large-scale population-based study UK-Biobank showed that higher LV mass was associated with increasing body mass index (BMI) (Petersen et al., 2017), explaining the BMI 72% of the global LV mass variability. However, myocardial and torso volumes affect the QRS amplitude in opposite ways. While larger ventricular geometries result in larger R and S wave amplitudes due to the increase of excitable myocardial tissue (see Figure 3), larger torso volumes result in lower R and S wave amplitudes due to the larger distance between the ventricles and the electrodes (see Figure 4). These two opposite effects coexist and lead to an ascending-descending behavior of the R wave amplitude with BMI as observed in clinical data (Kurisu et al., 2015). More specifically, precordial lateral leads display a slight increase in R wave from underweight to normal weight subjects followed by an abrupt decrease in the R amplitude for obese subjects (Kurisu et al., 2015). Despite the role of heart and body sizes, heart diseases and clinical conditions also affect QRS amplitude. Low QRS amplitude has been associated to different clinical conditions such as large infarct sizes (Katragadda et al., 2017), or to an increased risk of mortality in individuals free of apparent cardiovascular diseases (Usoro et al., 2014), while increased QRS amplitude has been associated to diseases such as left ventricular hypertrophy. Our study provides digital evidence in agreement with those findings and allows a systematic analysis of the effect of both ventricular and torso volumes in QRS amplitude.

### Heart Rotation and Position Mainly Affect QRS Morphology in Leads V1 to V4

The independent effect of heart position and orientation on QRS complex has also been investigated in the different heart-torso geometries showing slight differences in QRS duration with rotation around LA and LR axis directions and translation in the lateral and cranio-caudal directions (Figures 6, 7). Large morphological and amplitude changes of QRS in V1 to V3 leads are observed with rotation around the LA direction, whereas V1 to V5 are mainly affected by rotation around LR direction. QRS morphologies in V1 to V4 are mainly affected by translation of the heart within the torso in the lateral and cranio-caudal directions. This is in agreement with studies reporting that variations in the magnitude of the electrocardiogram as observed through leads placed on the anterior thorax are dominated by the solid angle (van Oosterom et al., 2000). Small rotation angles in the range of ±10° could be considered as mimicking pose changes caused by respiration (McLeish et al., 2002; Shechter et al., 2004). These resulted in QRS morphological changes mainly in the anterior leads V3 and V4, with very minor scaling amplitudes in V1 to V4 (see Supplementary Material S6) in agreement with (Carey et al., 2016). Large changes in heart position and orientation lead to remarkable changes in QRS morphology and amplitude in agreement with body position changes that significantly affect the ECG (Mincholé et al., 2014).

## Limitations and Future Work

The QRS complex reflects the ventricular depolarization and its pattern is determined by the electrical activation sequence of the ventricles, as well as the geometric relationship between the heart and the body surface anatomy as shown in this study. The activation sequence is, in turn, related not only to ventricular geometry but also to Purkinje, myocardial conduction pathways, and, other tissue conductivities (Boineau and Spach, 1968; Keller et al., 2010). In this study, we focused on evaluating the role of anatomical ventricular/torso properties on the QRS complex. Further studies can evaluate the role of additional factors on the QRS complex such as for example variability in the Purkinje system (Wallman et al., 2014), myocardial conductivities, and disease conditions (Lyon et al., 2018b).

We have selected a wide range of ventricular and torso geometries with volumes from 75 to 170 cm^3^, and from 23 to 54 dm^3^, respectively. However, the total variability of control human geometries may not be represented by this dataset. The results showed in this study are not however, expected to change qualitatively with consideration of a larger dataset in healthy subjects. Considering disease conditions will include additional factors determining variability in QRS morphology (Lyon et al., 2018a,b).

We have tackled the reconstruction of the shape of the surface body by completing the sparse information obtained from the MRI with a statistical shape model of the body surface. The agreement of the reconstructed torso and the MRI contours is high (no more than few millimeters of discrepancy for all the cases). However, there are large areas where we have no information of the body shape and we rely on the shape plausibility provided by the statistical shape model. This may result in differences between the reconstructed torso and the real one, and, therefore, in differences in the simulated ECG. This is a limitation for the accuracy of the reconstruction pipeline, but does not invalidate the main message of the work, in which we evaluate scenarios with plausible anatomies without claiming personalization of the subjects.

The propagation of the electrical activity in the torso by means of the integration of dipole source density formulation is chosen in order to simplify the numerical complexity of the computations and to avoid re-meshing in each scenario. Although this method does not allow the inclusion of tissue inhomogeneities in the torso, previous studies (Ramanathan and Rudy, 2001; Geneser et al., 2008), and, our own evaluations (see Supplementary Figure 3) have suggested that differences in the QRS complex between homogeneous and heterogeneous torsos are minimal. The inclusion of vasculature close to the heart and all tissue inhomogeneities may result in differences in the QRS complex between homogeneous and heterogeneous torsos. For these inclusions, different numerical models should be chosen such as the coupled bidomain model by the finite element methods.

## Conclusion

A population of 265 human torso-ventricular models is constructed based on clinical MRI using a computational pipeline for image analysis, construction of MRI-based anatomical models and HPC electrophysiological simulations. The technology demonstrated here can further exploit clinical databases to evaluate the functional implications of MRI-extracted features (Lyon et al., 2018b). Analysis of ECG simulations demonstrates that ventricular anatomy mostly determines QRS morphology in limb leads I and II, whereas heart position within the torso determines QRS morphology in the precordial leads, and especially V1 to V4. QRS duration is mainly influenced by myocardial volume, whereas QRS amplitude increases with large ventricular volumes and decreases with larger torso volumes. The new insights provided here are expected to aid in the discrimination in clinical recordings between the contribution of each patient’s specific anatomy and those arising from their disease state, and to contribute to accelerating the development of individualized score metrics for risk stratification, and therefore an improved tailoring of their pharmacological and electrical therapy.

## Data Availability

The datasets generated for this study can be downloaded from http://www.cs.ox.ac.uk/ccs/home.

## Author Contributions

AM, EZ, VG, and BR contributed to the conception and design of the study. AM performed the electrophysiological simulations and ECG analysis. EZ performed the personalization of the geometrical models. RA acquired the MRI of the patients. AM and EZ worked on the analysis of the results and writing of the manuscript. All authors contributed to manuscript revision, and read and approved the submitted version.

## Conflict of Interest Statement

The authors declare that the research was conducted in the absence of any commercial or financial relationships that could be construed as a potential conflict of interest.
