# Supplementary material for: MRI-Based Computational Torso/Biventricular Multiscale Models to Investigate the Impact of Anatomical Variability on the ECG QRS Complex
Source: Front Physiol. 2019 Aug 27;10:1103. doi: 10.3389/fphys.2019.01103 (PMC6718559; doi:10.3389/fphys.2019.01103)
Supplement: Supplementary file 1 [file Table_1.docx]

Supplementary Material

MRI-based computational torso/biventricular multiscale models to investigate the impact of anatomical variability on the ECG QRS complex

Ana Mincholé^1*^, Ernesto Zacur ^2*^, Rina Ariga ^3^, Vicente Grau^2¥^, Blanca Rodriguez^1¥^

^1^ Department of Computer Science, University of Oxford, Oxford, United Kingdom
^2^ Institute of Biomedical Engineering (IBME), University of Oxford, Oxford, United Kingdom
^3^ Division of Cardiovascular Medicine, Radcliffe Department of Medicine, University of Oxford, Oxford, United Kingdom

* **Equal contribution first authors**

^¥^ **Equal contribution senior authors**

**Correspondence:**Ana Mincholé, PhD
ana.minchole@gmail.com

# Generation of virtual torso-ventricular models

The known high dimensionality of the parameters determining the heart anatomy makes challenging the evaluation of its dependence on the QRS complex. In this work, a population of realistic torso-ventricular geometries is used to explore the role of ventricular anatomy, torso anatomy and heart orientation on the 12-lead QRS complexes.

We considered a total of 265 combined torso-ventricles anatomical models to quantify the effect of ventricular and torso volumes, and heart position and orientation on the QRS complex. Initially, twenty-five human heart-torso models were generated by combining the bi-ventricular geometries and torsos (including corresponding heart orientations and positions) from five healthy subjects. Each torso geometry also includes information on the heart pose, which represents the location and orientation (attitude) of the heart. This torso-pose linking is supported by physical constraints since each torso anatomy does not allow any realistic heart position due to chest boundaries. In particular, more horizontal LV long axis orientation in the frontal plane are related to an increased BMI, thus a higher diaphragmatic arc (Engblom et al., 2004, 2005). Additionally, (D. Dougherty, 1970) reported a correlation between thorax geometry and the long-axis orientation. In virtualizing a new torso-ventricular geometry, the heart is spatially transformed from a canonical reference frame to the pose defined by the torso (see Supplementary Figure 1). The canonical referential frame has its origin at the LV endocardium centroid at the atrioventricular plane. The atrioventricular plane is parallel to the SAX view specified by the clinician and defines the x-y plane with the x-axis pointing in the direction defined by right-to-left ventricle centroids.


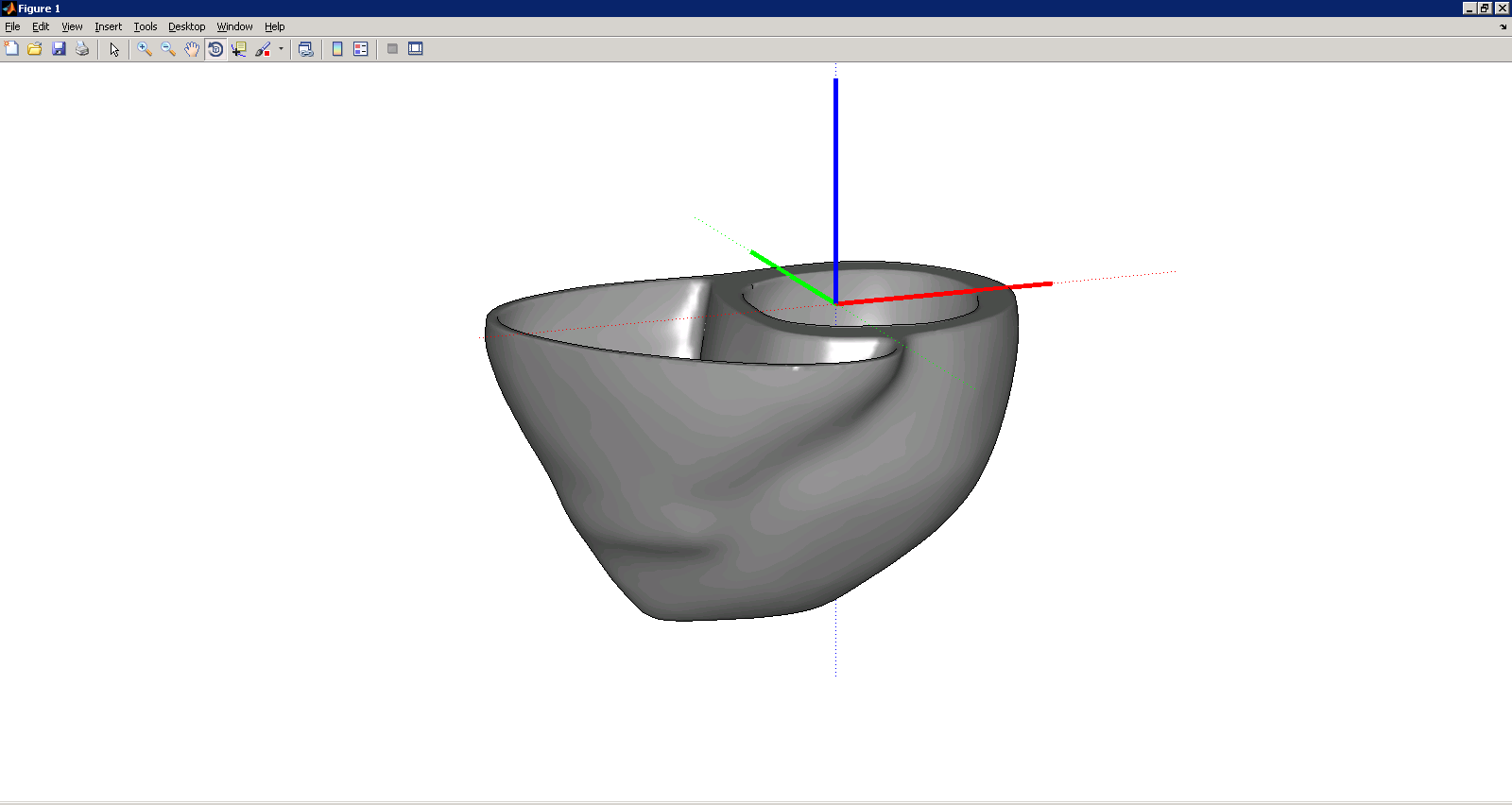

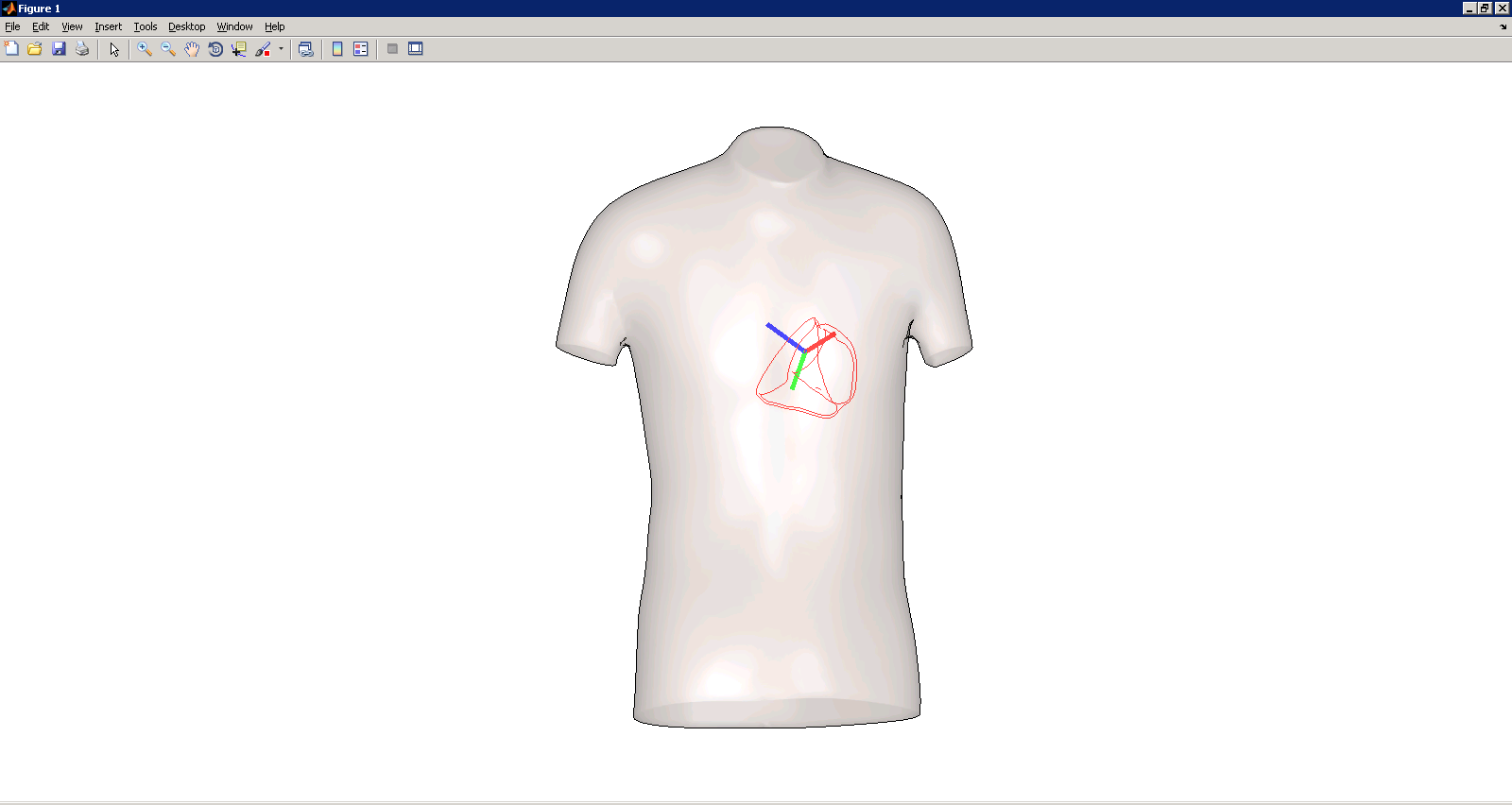


POSE

Canonical referential frame

**Supplementary Figure 1.** Heart pose within the torso defined as the transformation from a canonical coordinate system of the ventricular geometry to the torso coordinate system.

**Surface and Volumetric meshes for electrophysiological simulations**

All surfaces were remeshed with a restricted Frontal-Delaunay algorithm using the tool Jigsaw (Engwirda and Ivers, 2014), with different specified element sizes: 0.4 mm for the ventricular surfaces; 2 mm for the ribs; 3 mm for the lungs; and 10 mm for the body surface. These different element sizes were specified to ensure numerical convergence of the finite element software Chaste for electrophysiological simulations (Dutta et al., 2016; Pitt-Francis et al., 2009). Finally, tetrahedral volumetric meshes were constructed from these surfaces using Tetgen (Si, 2015), labelling each tetrahedron as a specific material based on the represented tissue.

# Electrophysiological simulations

The propagation of the electrical activity in the human ventricles and torso was modelled using the fully coupled heart-torso bidomain equations and solved with the Chaste software (Pitt-Francis et al., 2009). Human ventricular membrane kinetics were simulated with a modified version of the O'Hara-Rudy model 2011 (ORd) action potential model (O’Hara et al., 2011) published in (Dutta et al.).

Myocardial fibre structure was generated using a rule-based method to replicate the findings of Streeter (Streeter et al., 1969). Tissue conductivities were chosen to generate realistic conduction velocities for the choice of cell model, numerical scheme, and mesh resolution. For the myocardium, the three orthotropic intracellular conductivities were 1.5, 0.45, and 0.225 mS/cm in the fibre, sheet, and inter-sheet directions, respectively as in (Cardone-Noott et al., 2016). These conductivities resulted in conduction velocities of 50.0, 20.9, 13.2 cm/s measured from a 1D fibre model. The axisymmetric extracellular conductivities were 5.46, 2.03 and 2.03 mS/cm as in (Cardone-Noott et al., 2016). Isotropic conductivities of 0.39 and 0.20 mS/cm were assigned to the lung and bone elements, and 2.16 mS/cm in the rest of the torso as in (Cardone-Noott et al., 2016). Whereas previous studies have included additional structures (Keller et al., 2010), the importance of tissue inhomogeneities on the ECG is controversial and we thus chose to include the basic structures.

The activation model described in (Cardone-Noott et al., 2016), included 7 earliest activation sites (see Supplementary Figure 2), four in the LV (LV mid septum, LV basal anterior, and two LV mid-posterior) and three in the RV (RV mid septum, two RV free wall). These locations of the root nodes were mapped in each of our geometries to anatomically homologous locations from the geometry used in (Cardone-Noott et al., 2016). To model the tightly-packed endocardial Purkinje network, which allows a quick progression of the excitation in the endocardial layer from the sites of earliest activation, we used the Dijkstra's algorithm (Cardone-Noott et al., 2016). The endocardial activation speed was set to 120 cm/s to yield a distribution of endocardial activation times in accordance with the reported ex vivo microelectrode recordings by (Durrer et al., 1970).


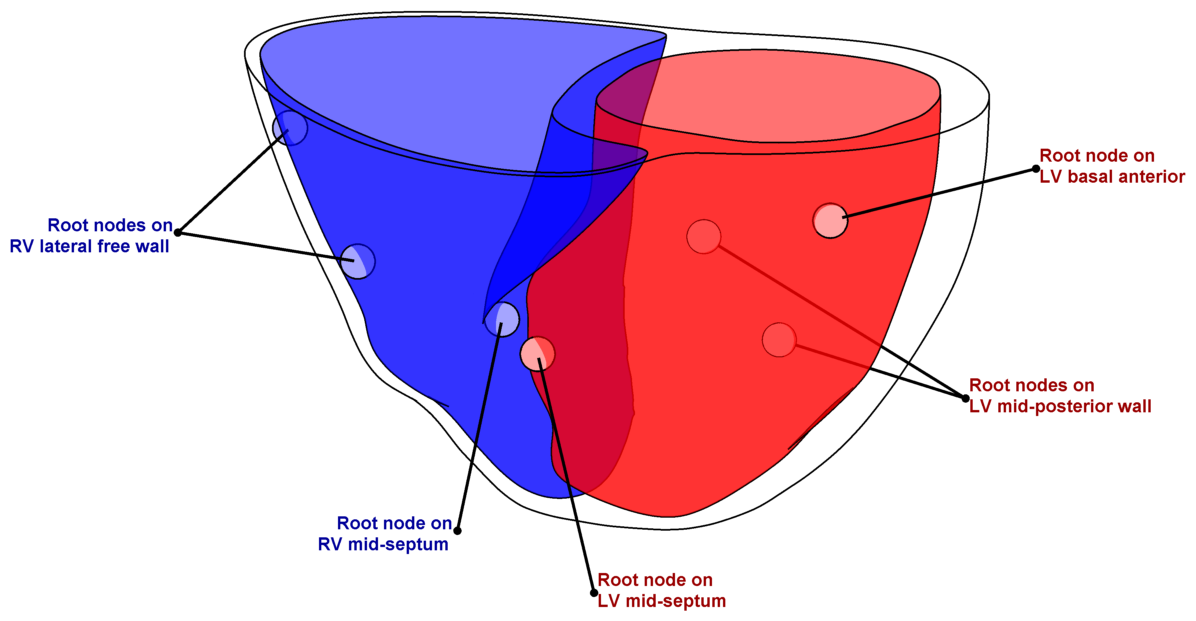


**Supplementary Figure 2.** Locations of the right (blue) and left (red) ventricular earliest activation sites.

Tissue electrophysiological heterogeneities were incorporated in our biventricular heart models. Transmural, apex to base, and interventricular cell electrophysiological heterogeneities based on experimental and clinical data from (Boukens et al., 2015; Drouin et al., 1995; Okada et al., 2011; Taggart et al., 2001) were incorporated. Apex-to-base heterogeneities were modelled by including a gradual increase of I_Ks_ conductance from base to apex resulting in APD differences of 40 ms. Transmural heterogeneities were modelled using layers of endocardial (45% of the transmural width), mid-myocardial (25%) and epicardial cells (30%) with different AP properties as in (O’Hara et al., 2011). These gradients resulted in interventricular APD differences of 25 ms between left and right ventricles.

Bidomain simulations coupled with the Poisson equation model to propagate the electrical activity up to the body surface were compared to the dipole model (Gima and Rudy, 2002) (see Eq. 1 in the main manuscript) yielding very similar QRS complexes and identical QRS durations, as shown in Supplementary Figure 3. Note that QRS amplitudes are not correct when using the dipole approach so we have used normalized factors for limb and precordial leads.

**Supplementary Figure 3.** Simulated ECG using the Poisson equation (solid black line) for the torso propagation and using the dipole model (solid grey line).

# Effect of ventricular geometry on the QRS complex

Supplementary Figure 4 illustrates the effect on the 12 lead ECGs of different ventricular geometries within the same torso-pose for each of the five subjects.


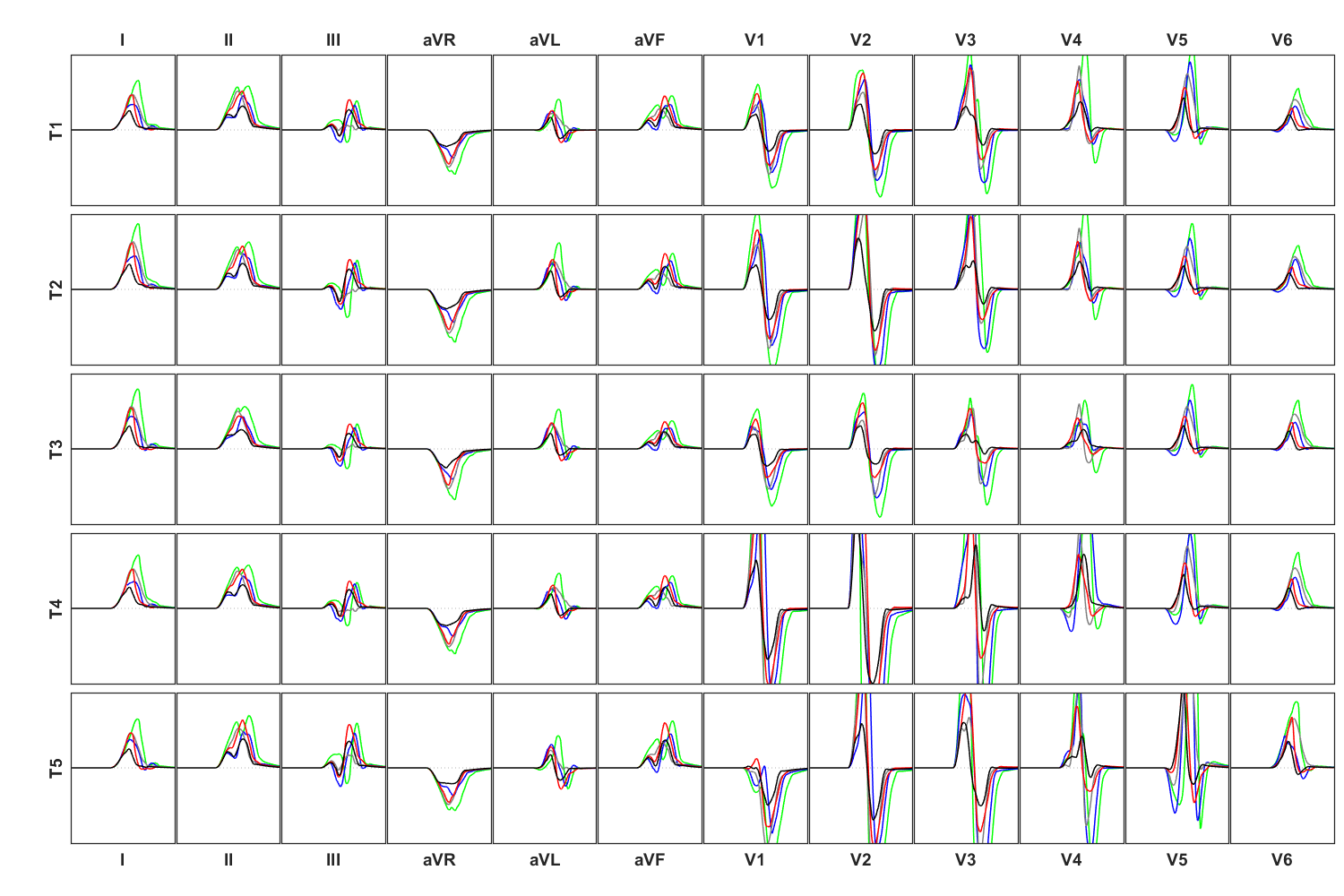


**Supplementary Figure 4.** Simulated QRS complexes obtained for five ventricular geometries placed in each of the five torso-poses (T1 to T5).

# Relationships between QRS duration and myocardial and torso volumes

Relationships between QRS duration and ventricular myocardial volume for each of the leads is shown in Supplementary Figure 5.

**Supplementary Figure 5.** Relationship between the myocardial volume and QRS duration for the ventricular geometries when placed in the five torso-poses (T1 to T5).

Relationships between QRS durations and torso volumes for each of the leads is shown in Supplementary Figure 6.

**Supplementary Figure 6.** Relationship between the torso volume and QRS duration for the ventricular geometries (H1 to H5) when placed in the five torso-poses.

# Warped QRS complexes

Supplementary Figure 7 shows the QRS morphologies from the 25 torso-ventricular geometrical models in two representative leads, aVL and V1. Additionally, the overlapping QRS complexes and the group-wise warped QRS complexes from which PC* is computed are illustrated in Supplementary Figure 7 and referred to as ‘Gather’ and ‘Warped’, respectively. The values reported in the top-right corner of each ‘Warped’ subplot represent the minimum obtained PC among all the possible pairs of comparisons after all the QRS complexes were group-wise warped to maximize that the similarity between them.

In Supplementary Figure 7-A, QRS complexes in lead aVL from different anatomical models sharing the same ventricular geometry display similar morphologies (see the different rows). On the contrary, QRS complexes sharing the same torso-pose display morphological differences (see the different rows). The minimum value for fixed heart geometries is PC*=0.87, and corresponds to H4, while for fixed torso-poses is PC*=0.67 and corresponds to T5. These qualitative and quantitative findings suggest that for lead aVL heart geometry has a larger influence on QRS morphology than torso-pose.

On the contrary, Supplementary Figure 7-B illustrates increase variability in QRS morphologies for models sharing the same ventricular geometry (see the different rows) while similar QRS morphologies are found for those sharing the same torso-pose (see the different columns). Accordingly, quantitative results show larger similarity measures for torso poses with PC*=0.74 than for heart geometries (PC*=0.41).

| **A. Lead aVL**  **** | **B. Lead V1**  **** |
| --- | --- |

**Supplementary Figure 7.** Simulated QRS morphologies for the five ventricular geometries (H1 to H5) when placed in the five torso-poses (T1 to T5) for leads aVL (Panel A) and V1 (Panel B). The overlapped QRS complexes derived from different hearts in one torso-pose (column) or from one heart in the different torso-poses (row) are displayed in (Gather) and this is followed by the warped QRS complexes without the effect of width and amplitude. In the warped subpanels, we compute the group-wise similarity measurement PC* that is displayed in the upper right corner.

# QRS Amplitude

Supplementary Figure 8 illustrates the QRS amplitude obtained from the 265 simulations conducted to evaluate the effect of rotation around the long axis and left-to-right ventricle directions, and translation along the lateral and cranio-caudal directions of the ventricles within the torso in the QRS complex. Note that QRS amplitudes are normalized with respect to that from the original heart position.


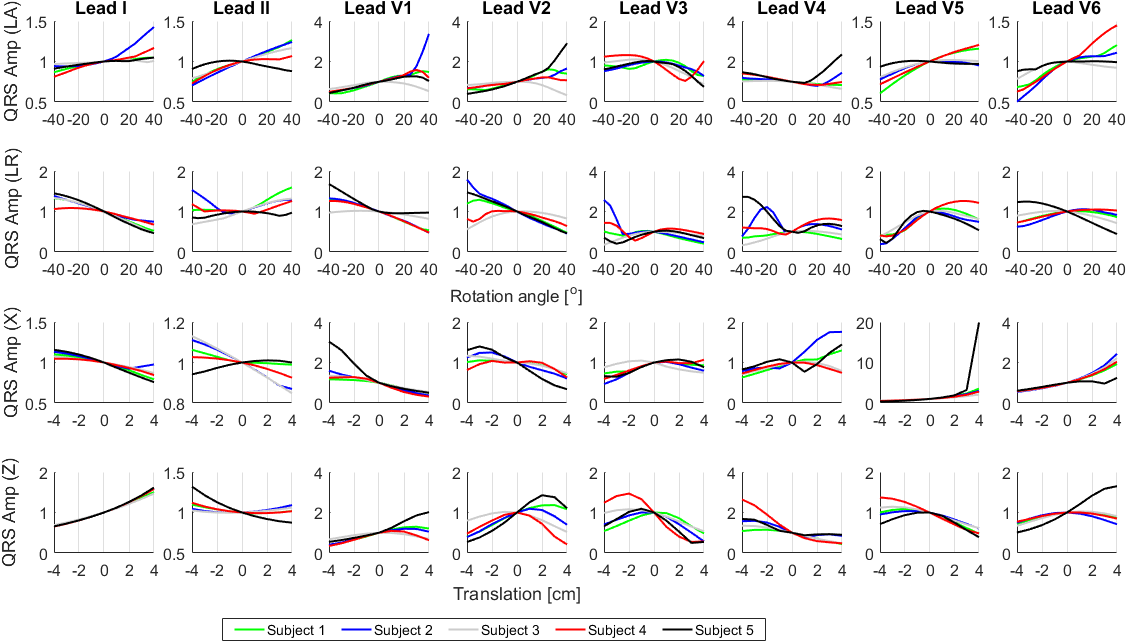


**Supplementary Figure 8.** QRS amplitude normalized by the QRS amplitude in the original pose for each lead (I, II, V1-V6) when the heart is rotated along the long axis (LA) and the left to right (LR) and translated in the lateral (X) and cranio-caudal (Z) directions.

# Effect of endocardial speed and myocardial conductivities on the contribution of the ventricular anatomy and the torso-pose on the QRS morphology of each of the leads

Ventricular geometry, and not the torso-pose, mainly determines the QRS morphology in the limb leads I and II, augmented leads, and V5. On the other hand, QRS morphology in septal and anterior leads V1 to V4, III and V6 is mostly determined by the torso anatomy and its linked heart positioning (see Figure 5 in the main manuscript). We have also evaluated the effect of endocardial and myocardial speed on the contribution of the ventricular anatomy and the torso-pose on the QRS morphology. Supplementary Figure 9 shows the QRS similarity measurement PC* of the simulated QRS morphologies under increased endocardial activation speed set to 180 cm/s and intracellular and extracellular conductivities increased 2-fold leading to similar results to Figure 5 in the main manuscript.

Variability in endocardial activation speed directly impacts the full ventricular activation sequence, affecting the duration and amplitude of the reconstructed QRS complexes but not QRS morphology. Variability in myocardial conductivities (as modelled by globally scaled intracellular and extracellular conductivity tensors) mainly affect the amplitude and duration of the QRS complex as shown in (Cardone-Noott et al., 2016). Therefore, results on the contribution of the ventricular anatomy and the torso-pose on the QRS morphology of each of the leads do not depend on the endocardial and myocardial conduction.


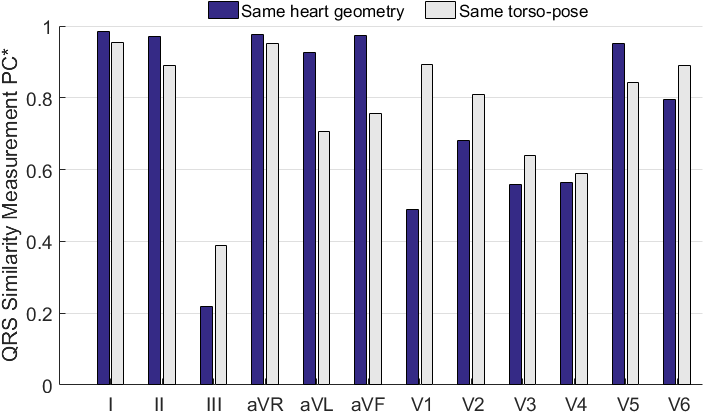


**Supplementary Figure 9.** Similarity measurement PC* of the simulated QRS morphologies when the heart geometry is fixed and the torso-pose varies (blue), and when the torso-pose is fixed and heart geometry varies (grey). Fast endocardial activation speed set to 180 cm/s and intracellular and extracellular conductivities increased 2 fold.

# References

Boukens, B. J., Sulkin, M. S., Gloschat, C. R., Ng, F. S., Vigmond, E. J., and Efimov, I. R. (2015). Transmural APD gradient synchronizes repolarization in the human left ventricular wall. *Cardiovasc Res* 108, 188–196. doi:10.1093/cvr/cvv202.

Cardone-Noott, L., Bueno-Orovio, A., Mincholé, A., Zemzemi, N., and Rodriguez, B. (2016). Human ventricular activation sequence and the simulation of the electrocardiographic QRS complex and its variability in healthy and intraventricular block conditions. *Europace* 18, iv4–iv15. doi:10.1093/europace/euw346.

D. Dougherty, J. (1970). The relation of the frontal QRS axis to the anatomic position of the heart * **. *Journal of electrocardiology* 3, 267–84. doi:10.1016/S0022-0736(70)80054-1.

Drouin, E., Charpentier, F., Gauthier, C., Laurent, K., and Le Marec, H. (1995). Electrophysiologic characteristics of cells spanning the left ventricular wall of human heart: evidence for presence of M cells. *J. Am. Coll. Cardiol.* 26, 185–192.

Durrer, D., van Dam, R. T., Freud, G. E., Janse, M. J., Meijler, F. L., and Arzbaecher, R. C. (1970). Total excitation of the isolated human heart. *Circulation* 41, 899–912.

Dutta, S., Mincholé, A., Quinn, T. A., and Rodriguez, B. Electrophysiological properties of computational human ventricular cell action potential models under acute ischemic conditions. *Progress in Biophysics and Molecular Biology*. doi:10.1016/j.pbiomolbio.2017.02.007.

Dutta, S., Mincholé, A., Zacur, E., Quinn, T. A., Taggart, P., and Rodriguez, B. (2016). Early afterdepolarizations promote transmural reentry in ischemic human ventricles with reduced repolarization reserve. *Prog Biophys Mol Biol* 120, 236–248. doi:10.1016/j.pbiomolbio.2016.01.008.

Engblom, H., Foster, J. E., Martin, T. N., Groenning, B., Pahlm, O., Dargie, H. J., et al. (2005). The relationship between electrical axis by 12-lead electrocardiogram and anatomical axis of the heart by cardiac magnetic resonance in healthy subjects. *American Heart Journal* 150, 507–512. doi:10.1016/j.ahj.2004.10.041.

Engblom, H., Hedström, E., Palmer, J., Wagner, G. S., and Arheden, H. (2004). Determination of the left ventricular long-axis orientation from a single short-axis MR image: relation to BMI and age. *Clinical Physiology and Functional Imaging* 24, 310–315. doi:10.1111/j.1475-097X.2004.00569.x.

Engwirda, D., and Ivers, D. (2014). Face-centred Voronoi Refinement for Surface Mesh Generation. *Procedia Engineering* 82, 8–20. doi:10.1016/j.proeng.2014.10.364.

Gima, K., and Rudy, Y. (2002). Ionic current basis of electrocardiographic waveforms: a model study. *Circ. Res.* 90, 889–896.

O’Hara, T., Virág, L., Varró, A., and Rudy, Y. (2011). Simulation of the Undiseased Human Cardiac Ventricular Action Potential: Model Formulation and Experimental Validation. *PLOS Computational Biology* 7, e1002061. doi:10.1371/journal.pcbi.1002061.

Okada, J., Washio, T., Maehara, A., Momomura, S., Sugiura, S., and Hisada, T. (2011). Transmural and apicobasal gradients in repolarization contribute to T-wave genesis in human surface ECG. *American Journal of Physiology - Heart and Circulatory Physiology* 301, H200–H208. doi:10.1152/ajpheart.01241.2010.

Pitt-Francis, J., Pathmanathan, P., Bernabeu, M. O., Bordas, R., Cooper, J., Fletcher, A. G., et al. (2009). Chaste: A test-driven approach to software development for biological modelling. *Computer Physics Communications* 180, 2452–2471. doi:10.1016/j.cpc.2009.07.019.

Si, H. (2015). TetGen, a Delaunay-Based Quality Tetrahedral Mesh Generator. *ACM Trans. Math. Softw.* 41, 11:1–11:36. doi:10.1145/2629697.

Streeter, D. D., Spotnitz, H. M., Patel, D. P., Ross, J., and Sonnenblick, E. H. (1969). Fiber Orientation in the Canine Left Ventricle during Diastole and Systole. *Circulation Research* 24, 339–347. doi:10.1161/01.RES.24.3.339.

Taggart, P., Sutton, P. M. I., Opthof, T., Coronel, R., Trimlett, R., Pugsley, W., et al. (2001). Transmural repolarisation in the left ventricle in humans during normoxia and ischaemia. *Cardiovascular Research* 50, 454–462. doi:10.1016/s0008-6363(01)00223-1.
